# The Novel Membrane-Bound Proteins MFSD1 and MFSD3 are Putative SLC Transporters Affected by Altered Nutrient Intake

**DOI:** 10.1007/s12031-016-0867-8

**Published:** 2016-12-16

**Authors:** Emelie Perland, Sofie V. Hellsten, Emilia Lekholm, Mikaela M. Eriksson, Vasiliki Arapi, Robert Fredriksson

**Affiliations:** 1 Unit of Functional Pharmacology, Department of Neuroscience, Uppsala University, Uppsala, Sweden; 2 Unit of Molecular Neuropharmacology, Department of Pharmaceutical Bioscience, Uppsala University, Uppsala, Sweden

**Keywords:** MFSD1, MFSD3, SLC, Protein expression

## Abstract

Membrane-bound solute carriers (SLCs) are essential as they maintain several physiological functions, such as nutrient uptake, ion transport and waste removal. The SLC family comprise about 400 transporters, and we have identified two new putative family members, major facilitator superfamily domain containing 1 (MFSD1) and 3 (MFSD3). They cluster phylogenetically with SLCs of MFS type, and both proteins are conserved in chordates, while MFSD1 is also found in fruit fly. Based on homology modelling, we predict 12 transmembrane regions, a common feature for MFS transporters. The genes are expressed in abundance in mice, with specific protein staining along the plasma membrane in neurons. Depriving mouse embryonic primary cortex cells of amino acids resulted in upregulation of *Mfsd1*, whereas *Mfsd3* is unaltered. Furthermore, in vivo, *Mfsd1* and *Mfsd3* are downregulated in anterior brain sections in mice subjected to starvation, while upregulated specifically in brainstem. *Mfsd3* is also attenuated in cerebellum after starvation. In mice raised on high-fat diet, *Mfsd1* was specifically downregulated in brainstem and hypothalamus, while *Mfsd3* was reduced consistently throughout the brain.

## Introduction

Membrane-bound transporters are physiologically important as they keep the homeostasis of soluble molecules within cellular compartments, and it is crucial to study their basic histology and function to understand the human body. The solute carrier (SLC) superfamily is the largest group of membrane-bound transporters in human and it includes 395 members, divided in 52 families (Hediger et al. 2004). SLCs utilize ATP-independent mechanisms to move nutrients, ions, drugs and waste over lipid membranes, and SLC deficiencies are associated with several human diseases (Hediger et al. 2013; Lin et al. 2015).

SLC proteins are grouped into Pfam clans based on functional domains, where the two largest clans, the major facilitator superfamily (MFS) and the amino acid-polyamine organocation (APC) clan (Hoglund et al. 2011), contain more than half of all SLC families. The MFS clan includes 16 SLC families, and all the MFS domain (MFSD)# proteins. Here, we are studying two novel MFSD# proteins, major facilitator superfamily domain containing 1 (MFSD1) and 3 (MFSD3). In humans, MFSD# transporters are commonly referred to as atypical SLCs, since they share high sequence similarity and evolutionary history with SLCs (Fredriksson et al. 2008; Perland et al. 2016; Sreedharan et al. 2011). MFSD# proteins are usually identified from large-scale genome annotation projects (Fredriksson et al. 2008) and named according to the HUGO Gene Nomenclature Committee (HGNC) nomenclature (Gray et al. 2016). At present, there are 17 MFSD# entries in the HGNC database and most are still orphans regarding expression, substrate and/or mechanism. However, MFSD2a was recently characterized as an omega 3 fatty acid transporter, located in endothelial cells in the blood-brain barrier in mice (Nguyen et al. 2014), MFSD4B is a sugar transporter in rat kidneys (Horiba et al. 2003) and human MFSD10 transport organic anions over the plasma membrane (Ushijima et al. 2008). MFSD7 (SLC49A3) is already classified into the SLC49 family based on sequence identity (Khan and Quigley 2013), but its expression and substrate is unknown. Furthermore, MFSD5 and MFSD11(Perland et al. 2016) are located in the plasma membrane (Perland et al. 2016), where they are suggested to be involved in regulating energy homeostasis (Perland et al. 2016). MFSD8 is expressed in lysosomal membranes (Damme et al. 2014; Siintola et al. 2007), and the messenger RNA (mRNA) of *Mfsd1* is expressed in various rat organs (Sreedharan et al. 2011). Looking at molecular-level information from the Kyoto Encyclopaedia of Genes and Genome (Kanehisa et al. 2016), MFSD1 is a predicted sugar transporter and MFSD3 a predicted acetyl-CoA transporter (Kanehisa et al. 2016).

Here, we present phylogenetic, homologic and histological data of MFSD1 and MFSD3. A phylogenetic tree was built to visualize the relationship between MFSD1 and MFSD3 and SLCs of MFS type, and global alignments were run against their evolutionary most closely related SLCs to investigate possible family affiliations. Secondary and tertiary protein models were built to study their transporter possibilities. Immunohistochemistry was performed to determine where the protein expression of MFSD1 and MFSD3 is in mouse brain, with focus on cell type specificity and subcellular location. Furthermore, we studied how nutrient availability affected the gene levels, both in mouse brain following starvation and high-fat diet (HFD) and in primary embryonic cortex cells following partial amino acid starvation.

## Material and Methods

### Phylogenetic Analysis

Human SLC amino acid sequences of MFS type (SLC2, 15, 16, 17, 18, 19, 22, 29, 33, 37, 40, 43, 45, 46 and SLCO), were downloaded together with human proteins including the MFS motif (MFSD1, MFSD2a, MFSD2b, MFSD3, MFSD5, MFSD7, MFSD8, MFSD10, MFSD11, MFSD13, SV2A, SV2B, SV2C, SVOP and SVOPL) from (Cunningham et al. 2015) (Ensembl release 84). MFSD1 and MFSD3 orthologous sequences were also obtained from the ENSEMBL database (listed in Table 1). These sequences were combined into a multiple PSI/TM sequence alignment using tcoffee (Notredame et al. 2000). The phylogenetic relationships between the sequences were inferred using the Bayesian approach as implemented in mrBayes 3.2.2 (Huelsenbeck and Ronquist 2001; Ronquist et al. 2012) to obtain the tree. The analysis was run via the Beagle library (Ayres et al. 2012) on an NVIDA 980Ti graphics card, and it was run on six chains (five heated and one cold) with two runs in parallel (*n* runs = 2) under the mixed amino acid model with eight gamma categories and invgamma as gamma rates for a total of 2,000,000 generations.

**Table 1:** MFSD1 and MFSD3 orthologue sequences identified in Ensembl (version 84) (Cunningham et al. 2015)

Species	MFSD1		MFSD3	
	Annotated name	Original sequence accession	Annotated name	Original sequence Accession
*A. carolinensis*	MFSD1	ENSACAT00000006961	MFSD3	ENSACAT00000010595
*D. melanogaster*	CG8602	FBtr0076884	–	–
	CG12194	FBtr0332476	–	–
*D. rerio*	mfsd1	ENSDART00000163827	MFSD3	ENSDART00000144995
*G. gallus*	MFSD1	ENSGALT00000015632	–	–
*G. aculeatus*	mfsd1	ENSGACT00000013516	–	–
*M. musculus*	Mfsd1	ENSMUST00000029344	Mfsd3	ENSMUST00000019224
*T. rubripes*	mfsd1	ENSTRUT00000009506	–	–
*X. tropicalis*	MFSD1	ENSXETT00000060684	–	–

### Sequence and Homology Modelling

To investigate the hypothesis that MFSD1 and MFSD3 indeed were SLCs, we retrieved the human MFSD1 (ENST00000415822.6) and MFSD3 (ENST00000301327.4) protein sequences and performed a prediction of their transmembrane sequences (TMS) using TMHMM server (v. 2.0; http://www.cbs.dtu.dk/services/TMHMM/). We then compared the TMS prediction with 3D homology models, to evaluate their possibility to actually span membranes; each amino acid in the membrane helices was identified and compared between the secondary and tertiary structure. The Swiss model fully automated homology program (Biasini et al. 2014) first identified MFSD1 and MFSD3 as MFS transporters and subsequently used the glycerol-3-phosphate MFS transporter (Huang et al. 2003) as template for MFSD1, and the YajR MFS transporter (Jiang et al. 2013) as template for MFSD3, when building the homology models. The tertiary protein structures were finalized using Swiss-Pdb Viewer (Guex and Peitsch 1997).

Finally, we investigated the sequence similarities between MFSD1 and MFSD3 and their phylogenetically closest relatives, the SLC29 and SLC33 family, respectively, using a global pairwise sequence alignment, using the Needleman-Wunsch algorithm (Li et al. 2015).

### Animal Handling

All experiments including mice were approved by the local ethical committee in Uppsala (Uppsala Djurförsöksetiska Nämnd, Uppsala district court) (Permit Number C419/12, C39/16 and C67/13) and conformed to the guidelines of European Communities Council Directive (2010/63) prior execution. Adult C57Bl6/J mice (Taconic M&B, Denmark) were used. All mice had free access to water and standard R3 chow (Lantmännen) unless otherwise stated. They were housed in a temperature-, light- and humidity-controlled room and euthanasia was performed during the light period by either cervical dislocation or transcardiac perfusion.

### Tissue Isolation, RNA Extraction and cDNA Synthesis from Wild-Type and Food-Restricted Mice

Material for an RNA panel including peripheral and central organs from adult mice was previously made (Perland et al. 2016) and subsequently used herein. Males were used for the panel with the addition of females for collection of female genitals. Additional mice were subjected to (1) normal chow, (2) 24-h starvation or (3) high-fat western diet (R368, Lantmännen) for 8 weeks before euthanasia, all according to (Perland et al. 2016). There was a 38 ± 9 % increase in weight in the obese group compared to a 12 ± 2.3 % increase in controls. *n* = 4 per diet for the specific brain areas: brainstem cortex, cerebellum, hypothalamus and striatum. The remaining brains (*n* = 6 for controls, *n* = 4 for starved and *n* = 6 in the HFD) were cut into larger brain sections using a brain matrix (Alto, 1 mm).

Briefly, mice were euthanized by cervical dislocation and collected tissues were preserved in RNA-later (QIAGEN) and the tissues were homogenized using 1 mm RNAse-free glass beads and a bullet blender (Averill Park, USA). The RNA was extracted from individual samples using Absolutely RNA Miniprep Kit (Agilent Technologies) following manufacturer’s instructions. A Heraesus Fresco 21 centrifuge was used at maximum speed at room temperature (RT) to remove debris by prefilter spin cups followed by RNA collection in binding cups. The RNA was washed and finally eluted. Concentrations were measured using an ND-1000 spectrophotometer (NanoDrop Technologies). The Applied Biosystems High-Capacity RNA-to-cDNA kit (Invitrogen) was then used for complementary DNA (cDNA) synthesis following manufacturer’s instructions. Two-microgram RNA was added to each cDNA synthesis reaction. The reaction was performed at 37 °C for 1 h, followed by 5 min incubation at 95 °C. cDNA from the same sample, but from different animals, were pooled and diluted to 5 ng/μl RNA in sterile water.

### Primers and Quantitative Real-Time PCR

The tissue distribution of *Mfsd1* and *Mfsd3* were determined using quantitative real-time PCR (qPCR). Primers were designed using Beacon Design 8 (Premier Biosoft, Palo Alto). For sample amplification: *Mfsd1* forward 5′-gacctctgtaaggatctg-3′, reverse 5′-tgctataatacaaaggaaagg-3′ and *Mfsd3* forward 5′-atttctggtcccagtgtg-3′, reverse 5′-gatgaacagtcagggtct-3. Reference genes: glyceraldehyde-3-phosphate dehydrogenase (*Gapdh*) forward 5′-gccttccgtgttcctacc-3′, reverse 5′-gcctgcttcaccaccttc-3′; beta tubulin 4B (*bTub*) forward 5′-agtgctcctcttctacag-3′, reverse 5′-tatctccgtggtaagtgc-3′; ribosomal protein L19 (*Rpl19*); forward 5′-aatcgccaatgccaactc-3′, reverse 5′-ggaatggacagtcacagg-3′; histone cluster 1 (*H3a*) forward 5′-ccttgtgggtctgtttga-3′, reverse 5′-cagttggatgtccttggg-3′; peptidylpropyl isomeras A (*Cyclo*) forward 5′-tttgggaaggtgaaagaagg-3′, reverse 5′-acagaaggaatggtttgatgg-3′; and actin-related protein 1B (*Actb*) forward 5′-ccttcttgggtatggaatcctgtg-3′, reverse 5′-cagcactgtgttggcatagagg-3′.

One-microliter pooled cDNA (5 ng/μl RNA) was used per qPCR reaction. The qPCR mastermix contained 2× DreamTaq Buffer (Thermo Fisher Scientific), 0.2 μl 20 mM dNTP, 0.05 μl of both forward and reverse primer (100 pmol/μl), 0.5 μl of SYBR Green (1:10,000; Invitrogen) in TE buffer (pH 7.8), 1 μl dimethyl sulfoxide (Sigma-Aldrich) and 0.08 μl DreamTaq polymerase (5 U/μl, Thermo Fisher Scientific). The volume was adjusted to 20 μl with sterile water. The reactions were run on iCycler real-time detection instrument (Bio-Rad Laboratories) and followed these conditions: initial denaturation for 30 s at 95 °C followed by 45 cycles of 10 s at 95 °C, 30 s at 55–61 °C (optimal temperature for each primer pair) and 30 s at 72 °C. Thereafter, a melting curve was performed by increasing the temperature 0.5 °C per cycle, during 81 cycles at 10-s intervals, starting from 55 °C. All reactions were performed in triplicates, and negative controls were included on each plate. All experiments were repeated twice.

### Data Analysis and Relative Expression Calculations

CT values were obtained from the MyIQ (Bio-Rad Laboratories) software. Primer efficiencies were calculated using LinRegPCR software, followed by Grubbs test (GraphPad software) to remove outliers in the efficiency calculations. The GeNorm protocol (Vandesompele et al. 2002) was used to detect which reference genes were stable. The geometric means of the stable reference genes were used to normalize the expression of *Mfsd1* and *Mfsd3*. For the wild-type (wt) panel following reference genes were used: *Gapdh*, *bTub*, *Rpl19*, *Cyclo* and *Actb*. The samples from mice raised on various diets were normalized against these genes: *Gapdh*, *H3a* and *Actb*. The duplicates in experimental set ups were analysed separately, and the normalized mRNA levels ±SD were plotted. Unpaired *t* tests were performed using GraphPad Prism 5 between the control group and the starved group or the HFD group for each brain section/region. The significance levels were Bonferroni corrected for multiple testing and the significance levels was set to *p ≤ 0.0493, **p ≤ 0.00998 and ***p ≤ 0.001.

### Western Blot

A protein fraction from mouse brain was used as western blot samples prepared as described in (Perland et al. 2016). Proteins were mixed with Laemmeli Sample Buffer (Bio-Rad) and 2-mercaptoethanol (Fluka) and loaded onto a 4–15 % TGX Miniprotean gel (Bio-Rad). A pre stained molecular weight marker was used as reference (Thermo Fisher). The electrophoresis was run at 200 V to separate the proteins before blotted onto PVDF membrane (Immobilon-P, Millipore) at 4 °C at 50 V for 1 h. The membrane was blocked for 1 h in 5 % milk (Bio-Rad) diluted in TTBS prior primary antibody incubation at 4 °C overnight. Anti-MFSD1 (1:200, rabbit, cat. no: SAB3500575, Sigma-Aldrich) and Anti-MFSD3 (1:500, rabbit, cat. no: AV51707, Sigma-Aldrich) were used. The membranes were washed before incubated for 1 h at RT in secondary HRP coupled anti-rabbit (Invitrogen) or HRP coupled anti-goat (Invitrogen) antibodies diluted 1:10,000 in milk blocking solution. Development was done using Clarity Western ECL Substrate (Bio-Rad), and staining was visualized using a CCD camera (Bio-Rad).

### Brain Section Preparation

Wild-type adult male C57Bl6/J mice were anaesthetised with an intraperitoneal injection of 0.5 mg/g body weight pentobarbital sodium (Apoteksbolaget). Transcardiac perfusion was performed using phosphate-buffered saline (PBS) (137.0 mM NaCl, 2.70 mM KCl, 8.10 mM Na_2_HPO_4_) followed by 4 % formaldehyde (HistoLab) fixation. The brain was dissected and stored in 4 % formaldehyde overnight at 4 °C before further processing. Seventy-micrometer free-floating brain sections were prepared by washing the brain twice in Tris-buffered saline (TBS) (0.04 M Trizma HCl, 0.01 M Trizma base, 0.15 M NaCl, pH 7.4) for 5 min followed by embedding in 4 % agarose gel (Pronadisa Conda). Sections were cut with a vibratome Leica VT 1200S (Leica Microsystems). For 12-μm coronal cryostat sections, the brains were incubated in 30 % sucrose prior to freezing. Sections were cut in a cryostat (HM 560, Microm International).

### Non-fluorescent Staining on Free-Floating Mouse Brain Sections

All chemicals were purchased from Sigma-Aldrich, unless otherwise stated. Seventy-micrometer brain sections from two mice were rinsed in TBS 4 × 8 min before and after incubation in 10 % MeOH and 3 % H_2_O_2_ (Merck Millipore) in TBS for 10 min. Sections were incubated in 1 % blocking reagent (Roche Diagnostics) for 1 h followed by primary antibody incubation with anti-MFSD1 (1:1000) and anti-MFSD3 (1:1000) diluted in supermix (0.25 % gelatine, 0.5 % Triton X-100 in TBS) overnight at 4 °C. Sections were rinsed in TBS 2 × 1 + 4 × 8 min and incubated in secondary antibody (biotinylated goat-anti-rabbit IgG (H + L), Vector laboratories) diluted 1:400 in supermix for 1 h. Sections were rinsed in TBS 5 × 8 min before and after incubation in ABC kit (Reagent A, Reagent B (Vectastain, Vector Laboratories) and diluted 1:800 in supermix for 1 h. Sections were incubated in 0.05 % 3.3 diaminobenzidine tetrahydrochloride, 0.35 % NiCl and 0.015 % H_2_O_2_ and rinsed 4 × 5 min in TBS before mounted on gelatinized microscope slides (Menzel Gläser). Samples were dehydrated in 70 and 95 % EtOH for 5 min, 100 % EtOH (Solveco) for 10 min prior xylene incubation for 20 min. Slides were mounted in DPX Mounting for histology with micro cover slides Superfrost Plus (Menzel Gläser). Sections were analysed using a Mirax Pannoramic midi scanner with the Pannoramic Viewer software version 1.15.4 RTM (3dHistech).

### Immunohistochemistry on Embryo and Adult Brain Sections

Fluorescent immunohistochemistry was performed on cryostat-sectioned brains. Sections were washed in PBS followed by primary antibody incubation overnight. All antibodies were diluted in supermix. Anti-MFSD1 (1:50) and anti-MFSD3 (1:50) were used to label the proteins. They were co-stained with the neuronal nuclear marker anti-NeuN (1:400 mouse, cat. no: MAB377, Millipore) (Mullen et al. 1992), the astrocyte marker anti-GFAP (1:400 mouse, cat. no: MAB360, Millipore) (Reeves et al. 1989) and the dendritic marker anti-MAP2 (1:400 mouse, cat. no: M4403, Sigma-Aldrich) (Izant and McIntosh 1980). After additional PBS washes, slides were incubated with secondary antibody for 2 h at RT. Secondary antibodies used are as follows: alexa 488 goat-anti-rabbit, alexa 488 donkey-anti-goat and alexa 594 donkey-anti-mouse (Invitrogen) diluted 1:400. PBS washes followed by 10 min DAPI staining (1:3000 in PBS, Sigma-Aldrich) and additional washes before mounted in Mowiol anti-fade media (25 g Mowiol 4–88 in 100-ml 1× PBS, pH 8.0, 50 ml glycerol, 3 ml of 1 % thimerosal and 100 μg/ml *n*-propyl gallate in (Sigma-Aldrich)). Images were taken using an Olympus microscope BX53 with an Olympus DP73 camera. The micrographs were acquired by cellSens Dimension software.

### Immunocytochemistry on Embryonic Primary Cortex Cells

Embryos at days 14–15 were retrieved and their cortices were used to create embryonic primary cell cultures as described in (Perland et al. 2016). Briefly, pregnant females were euthanized by cervical dislocation followed by embryo retrieval. The cortices were dissected and dissociated using both chemical and mechanical procedures followed by filtration to ensure single cells before seeding the cells in plating media (DMEM-F12 (Gibco), 10 % FBS (Gibco), 2 mM L-glutamine (Invitrogen), 1 mM Na-pyruvate (Invitrogen) and 1 % penicillin/streptomycin (Invitrogen)) in a density of 7.5 × 10^4^ cells on poly-L-lysine (Sigma-Aldrich)-coated cover slips. Cells were incubated for 3 h at 37 °C, 5 % CO_2_ before the media was changed to growth media (Neurobasal-A (Gibco), 2 mM L-glutamine, 1 mM Na-pyruvate, 1 % penicillin/streptomycin and 2 % B27 (Invitrogen)). Seventy-five percent of the media was changed every 3–4 days and at day 10, the cells were fixed in 4 % formaldehyde for 1 h and rinsed in PBS.

Immunocytochemistry was performed as previously described in (Perland et al. 2016). Anti-MFSD1 (1:50) and anti-MFSD3 (1:50) were co-stained with: anti-PAN Neuronal marker (1:300, mouse, cat. no: MAB2300, Millipore); anti-Lamp2 (1:100, rat, cat.no: Ab13524, Abcam) (Chen et al. 1985) and anti-Golgi58K (1:100, goat, cat. no: Ab19072, Abcam) (Anthony and Gietzen 2013). MFSD1 was additionally stained together with anti-Synaptotagmin (1:100, mouse, cat. no: MAB5200, Millipore) (O’Connor and Lee 2002) and anti-synaptophysin (1:200, mouse, cat. no: Ab8049, Abcam) (McMahon et al. 1996). Images were taken using a LSM710 super resolution microscope (Zeiss) with the Black Zen software. Z-stacks were acquired and merged into 2D images using Fiji Image J (Schindelin et al. 2012; Schindelin et al. 2015).

### Amino Acid Deprivation on Primary Mouse Embryonic Cortex Cells

Starved primary cortex cells were deprived of the following amino acids: glycine, L-alanine, L-asparagine, L-glutamine, L-histidine, L-isoleucine, L-leucine, L-serine and L-valine. Control medium was prepared by adding 2.0 mM GlutaMAX to EBSS medium (Gibco, Life technologies) and glycine, L-alanine, L-arginine, L-asparagine, L-cysteine, L-histidine, L-isoleucine, L-leucine, L-lysine, L-methionine, L-phenylalanine, L-proline, L-serine, L-threonine, L-tryptophan, L-tyrosine and L-valine (Sigma-Aldrich) was added in the same concentrations as in the Neurobasal-A medium. Medium for the amino acid-deprived cells was prepared by adding L-arginine, L-cysteine, L-lysine, L-methionine, L-phenylalanine, L-proline, L-threonine, L-tryptophan and L-tyrosine (Sigma-Aldrich) in the same concentrations as in the Neurobasal-A medium to the Earle’s balanced salt solution (EBSS) medium. Both the control and starvation medium was supplemented with 1.0 mM sodium-pyruvate, 1 % penicillin/streptomycin, 2 % B-27® (50X), 4× MEM vitamin solution (100×) (Gibco, Life technologies) and 10.9 mM HEPES (1 M) buffer solution (Gibco, Life technologies). The experiment was run in triplicates in each treatment group. The cells were treated in the limited amino acid medium or the complete amino acid medium for 3, 7, or 12 h before RNA was extracted using RNeasy Midi Kit (QIAGEN), following the manufactures protocol. cDNA synthesis and qPCR were run and analysed as previously described. cDNA from the triplicates in each treatment group were pooled and the geometric mean of the expression of the housekeeping genes *Gapdh*, *H3a* and *Actb* were used for normalization. Unpaired *t* tests were run to calculate the differences in gene expression between the normally fed and the starved cells, *p ≤ 0.05, **p ≤ 0.01 and ***p ≤ 0.001 were used as significance levels. Normalized expressions ±SD were plotted.

## Results

### Phylogenetic Analysis Showed Clusters with SLCs

**Fig. 1:**
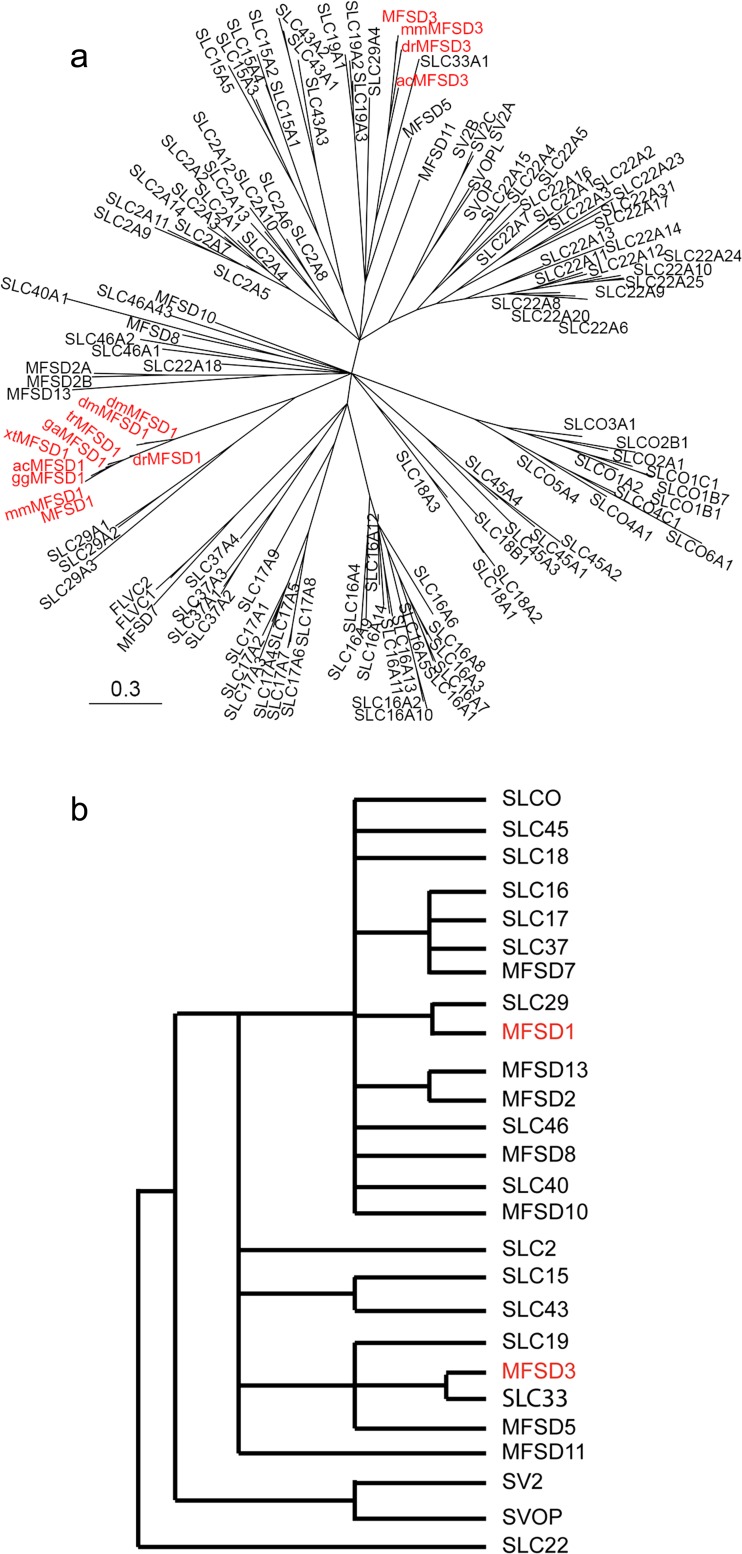
Evolutionary conservation of MFSD1 and MFSD3 and their sequence similarities to SLCs. **a** The phylogenetic tree was calculated using a Bayesian approach. Orthologues of MFSD1 and MFSD3 are marked in *red*. Species abbreviations: *ac Anolis carolinensis*, *dm Drosophila melanogaster*, *dr Danio rerio*, *ga Gasterosteus aculeatus*, *gg Gallus gallus*, *mm Mus musculus, tr Takifugu rubripes, xt Xenopus tropicalis.* The tree was used as base when creating the schematic representation of the branching order (**b**)

We inferred the phylogenetic relationship between plausible orthologues to human MFSD1 and MFSD3 using the Bayesian approach to obtain the tree in Fig. 1a. MFSD1 and MFSD3 were found to be present in chordates, whereas MFSD1 also was detected in *Drosophila melanogaster*. MFSD1 and MFSD3 also clustered with SLCs of MFS type and other MFSD proteins, suggesting they are atypical putative SLCs. Interestingly, MFSD1 was mostly related to SLC29 family members, whereas MFSD3 grouped together with SLC33 (Fig. 1a). However, note also that SLC29A4 diverged evolutionary from the other SLC29 members, since it clustered with the SLC19 family (Fig. 1a) and was thus more related to MFSD3, than MFSD1. In Fig. 1b, we depicted the schematic branching order between SLC families and how they interrelate with known MFSD proteins. From this, it is evident that MFSD1 is most closely related to the facilitative nucleoside transporter SLC29 family; while MFSD3 clusters with SLC33, the acetyl-CoA transporter family (Fig. 1b). Regarding the MFSD proteins, they phylogenetically group together with SLCs in an arbitrary order, enhancing the hypothesis that they, in fact, are membrane-bound transporters belonging to various SLC families.

### Secondary Structures and Sequence Identity with Known SLC Transporters

**Fig. 2:**
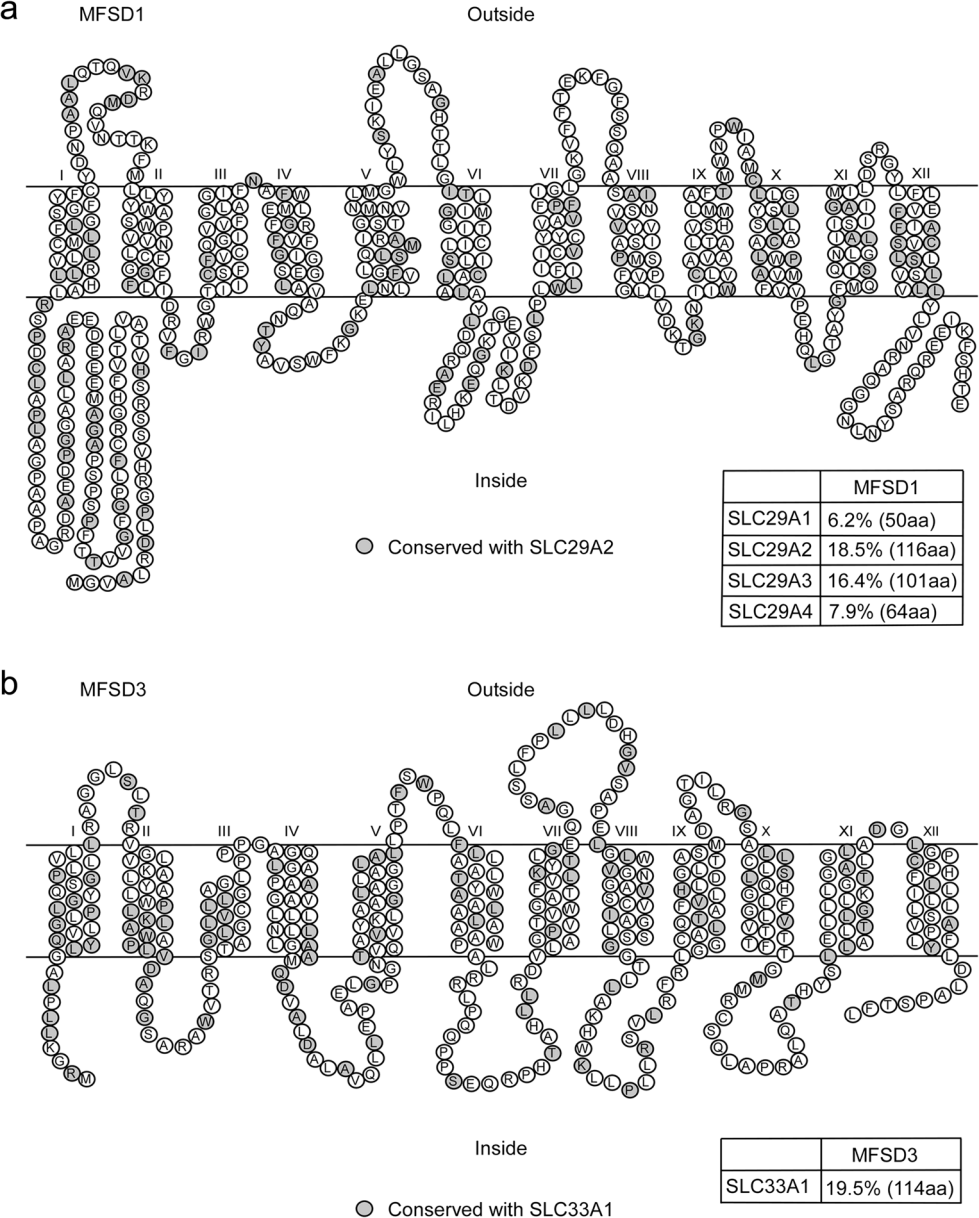
Secondary structures of MFSD1 and MFSD3. The amino acids in the MFSD1 (**a**) and MFSD3 (**b**) proteins are depicted as 12 TMS regions (*I*–*XII*). As MFSD1 and MFSD3 are closely related to the SLC29 and SLC33 family, we aligned the proteins to study sequence identities. The *grey circles* represent the identical amino acids after alignment between MFSD1 and SLC29A2 and MFSD3 with SLC33A1. The *tables* represent the sequence identities obtained from global pairwise alignments between MFSD1 and SLC29 members and MFSD3 and SLC33A, where the amino acids in brackets are the once identical between the proteins

The TMS prediction provided a probability plot, showing transmembrane helices, and it revealed 12 TMS for both MFSD1 and MFSD3 (Fig. 2a, b). All identified TMS did not meet the criterion for highest probability, suggesting that the amino acid sequences are diverging from the more common TMS structures. To compare the MFSD1 and MFSD3 proteins with known SLCs, we aligned their sequences with members of the phylogenetically closest family, SLC29 and SLC33, respectively. The largest MFSD1 splice variant shared following sequence identity with SLC29 family members; 6.2 % of the sequence was identical with SLC29A1, of which they shared 50 amino acids, 18.5 % with SLC29A2 (116 identical aa, depicted in grey in Fig. 2a), 16.4 % with SLC29A3 (101 identical aa) and 7.9 % with SLC29A4 (64 identical aa). MFSD3 was aligned with SLC33A1 (Fig. 2b.), displaying 19.5 % sequence identity, with 114 identical amino acids.

### Homology Models for MFSD1 and MFSD3

**Fig. 3:**
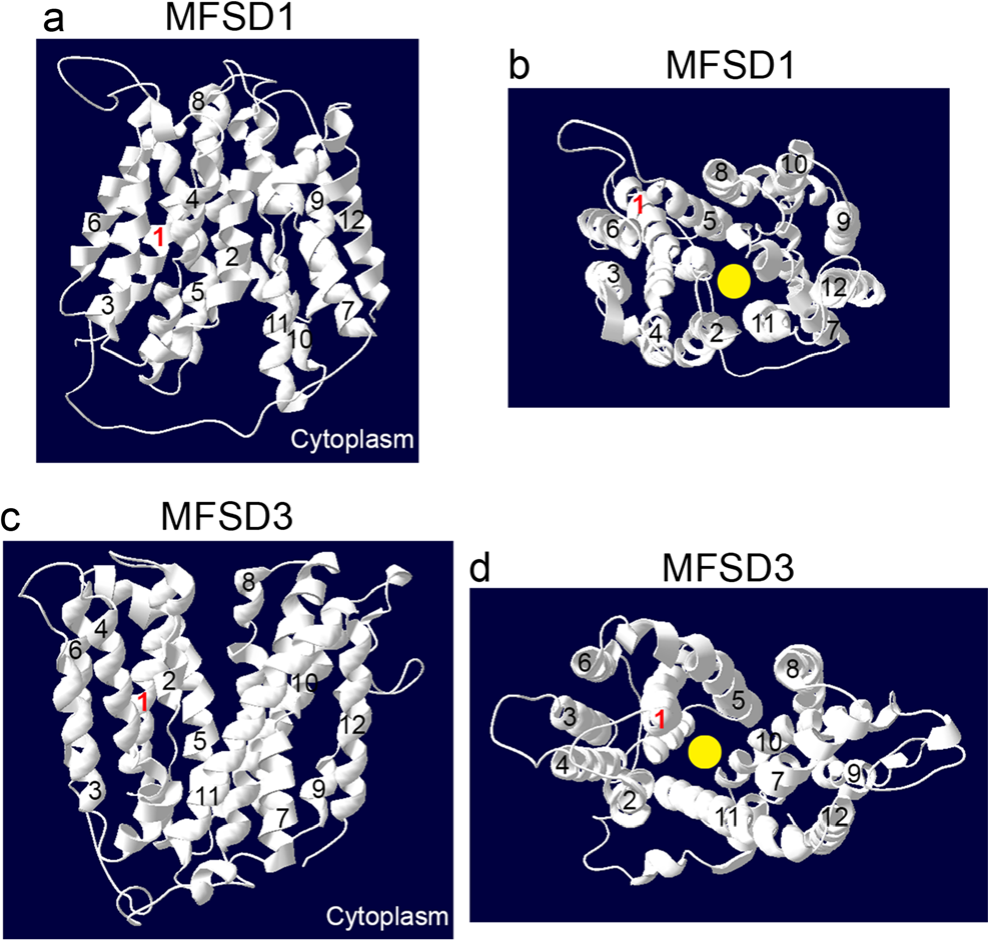
Homology modelling. The tertiary structures of MFSD1 (**a**, **b**) and MFSD3 (**c**, **d**) revealed 12 transmembrane regions, marked 1–12. **a**, **c** The side-views of the homology models. **b**, **d** The top-view, in which a possible substrate pore, marked in *yellow*, can be seen. MFS proteins were used as templates when building the homology models

Also with homology modelling, 12 possible transmembrane helices for both MFSD1 (Fig. 3a, b) and MFSD3 (Fig. 3c, d) were identified. The homology structures were aligned against MFS protein templates, where the MFSD1 model had a global model quality estimation (GMQE) value of 0.47 while the MFSD3 model had a GMQE value at 0.5. GMQE scale range from 0 to 1, to indicate the credibility of the models. Looking at the tertiary structures from top view (Fig. 3b, d), both proteins seemed to form a pore, through which a substrate possibly could be transported. Based on the modelling, both the N- and C-terminals were in the cytoplasm.

### RNA Expression in Wild-Type Mice

**Fig. 4:**
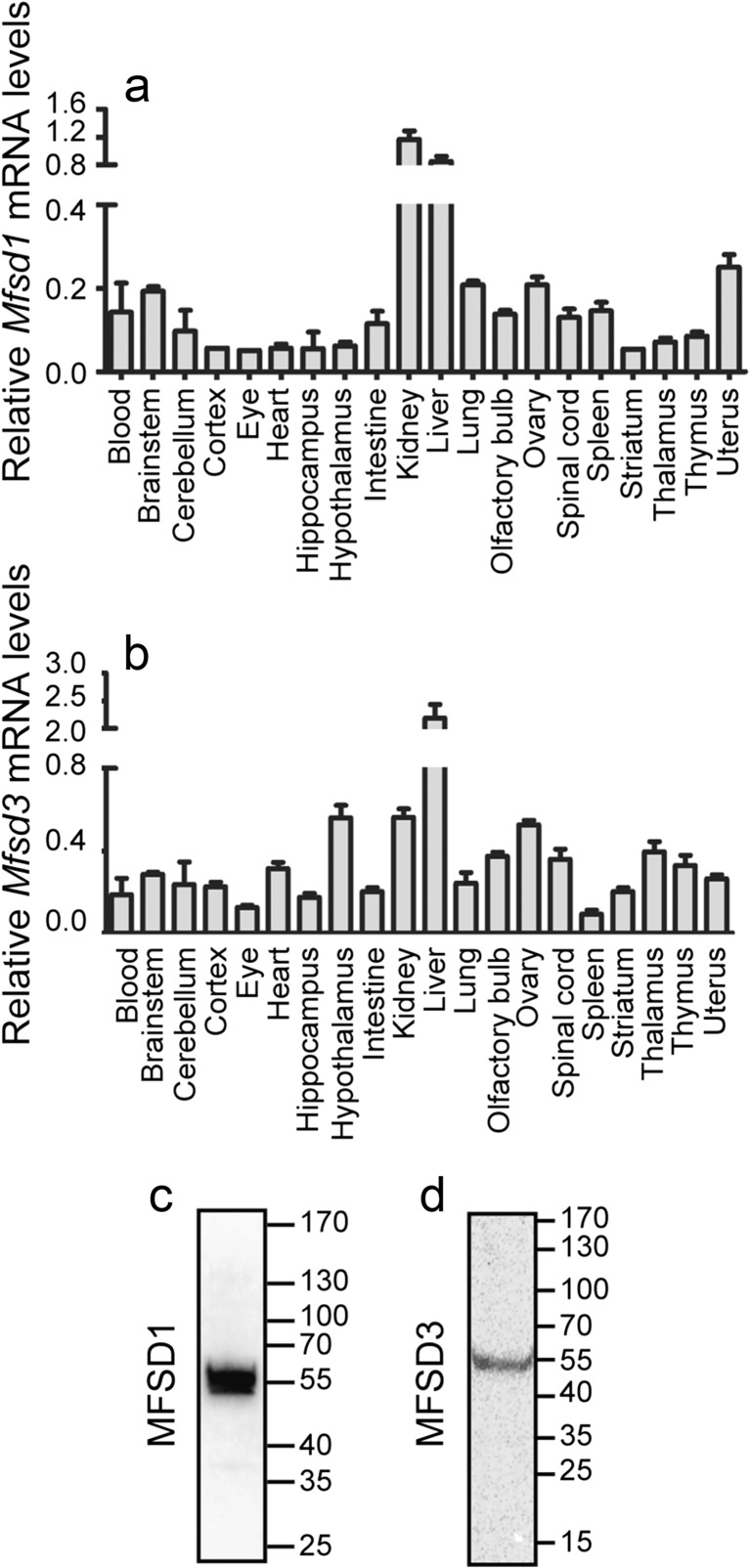
qPCR measurements of *Mfsd1* and *Mfsd3* in wild-type mice and antibody verification. The relative mRNA expression levels (normalized mean ± SD) of *Mfsd1* (**a**) and *Mfsd3* (**b**) in organs from C57BL/6 mice are displayed in the column charts. *n* = 5 per organ. For protein detection, commercially bought antibodies were used and their specificity was verified with western blot prior staining. Anti-MFSD1 bound proteins at size 58 kDa (**c**) and anti-MFSD3 revealed one band at size 50 kDa (**d**). The band sizes corresponded well with predicted protein sizes; hence it verified the antibody specificity

The gene expressions of *Mfsd1* and *Mfsd3* were analysed in organs from C57BL/6 mice, using qPCR. *Mfsd1* was detected throughout the panel, with high relative expression in peripheral organs such as kidney (1.17 ± 0.12) and liver (0.85 ± 0.08) (Fig. 4a). Of the central areas, highest expression was measured in brainstem (0.19 ± 0.01) and spinal cord (0.13 ± 0.02) (Fig. 4a). *Mfsd3* displayed abundant expression throughout the periphery and the CNS with high relative levels in peripheral organs such as liver (2.19 ± 0.25), kidneys (0.56 ± 0.04) and ovaries (0.53 ± 0.02) and central areas such as hypothalamus (0.56 ± 0.06), thalamus (0.40 ± 0.05) and spinal cord (0.36 ± 0.05) (Fig. 4b).

### Western Blot Verifies Antibodies Specificities

To verify the specificity of the commercially available antibodies, western blot was run on fractionated mouse brain. The anti-MFSD1 antibody bound to one band at size 58 kDA (Fig. 4c), which agrees well with the predicted mouse splice variant at 51 kDA (Ensembl no: ENSMUST00000029344). The anti-MFSD3 antibody bound proteins at approximate size 50 kDa (Fig. 4d) which corresponds to the mouse splice variant at size 43 kDA (Ensembl no: ENSMUST00000019224). Hence, both antibodies bound proteins at sizes within ±10 kDA from the predicted annotated size so the western blot verified the specificities of the antibodies.

### Bright Field Immunostaining to Screen for Protein Distribution

**Fig. 5:**
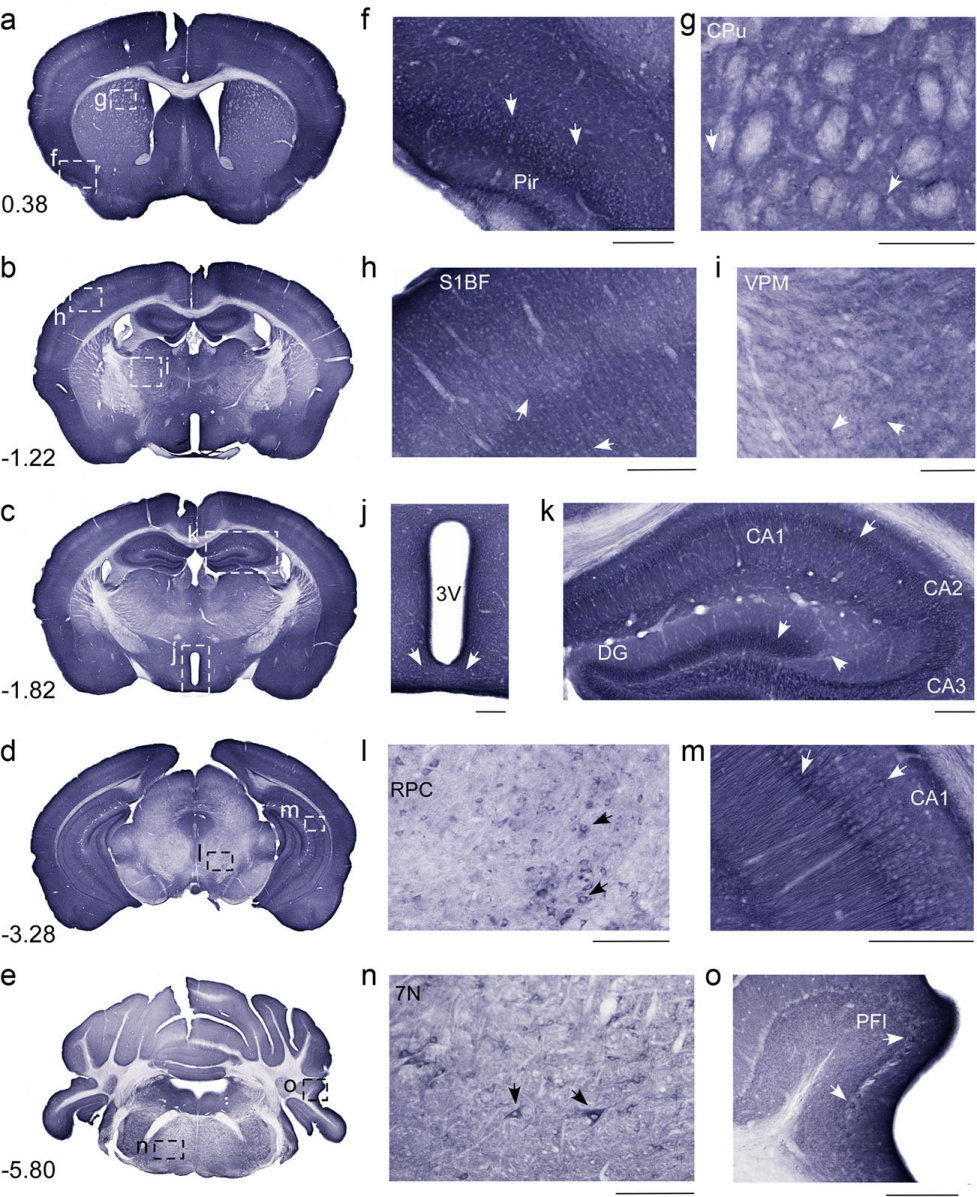
Bright field MFSD1 protein screening in coronal mouse brain sections. DAB immunohistochemistry for MFSD1 on free-floating mouse brain sections with overview pictures (**a**–**e**), close-up pictures with *scale bars* of 200 μm (**f**–**o**). The Bregma regions correspond to the mouse brain in (Franklin and Paxinos (2007). There are overview micrographs in which the magnifications are marked with dashed lines: (**a**) Bregma 0.38 mm, (**b**) Bregma −1.22 mm, (**c**) Bregma −1.82 mm, (**d**) Bregma −3.28 mm and (**e**) Bregma −5.80 mm. The magnified images are as followsed: (**f**) piriform cortex and (**g**) caudate putamen (striatum); (**h**) primary somatosensory barrel cortex and (**i**) ventral posteromedial thalamic nucleus; (**j**) the arcuate hypothalamic nucleus and (**k**) hippocampus fields CA1, CA2 and CA3. Large cells were seen in the red nucleus (**l**), both in the parvocellular and magnocellular part. Projections are depicted in the hippocampus region CA1 (**m**) and cells in the facial nucleus (**n**). Finally, the Purkinje cell layer in the cerebellum (**o**) is shown

**Fig. 6:**
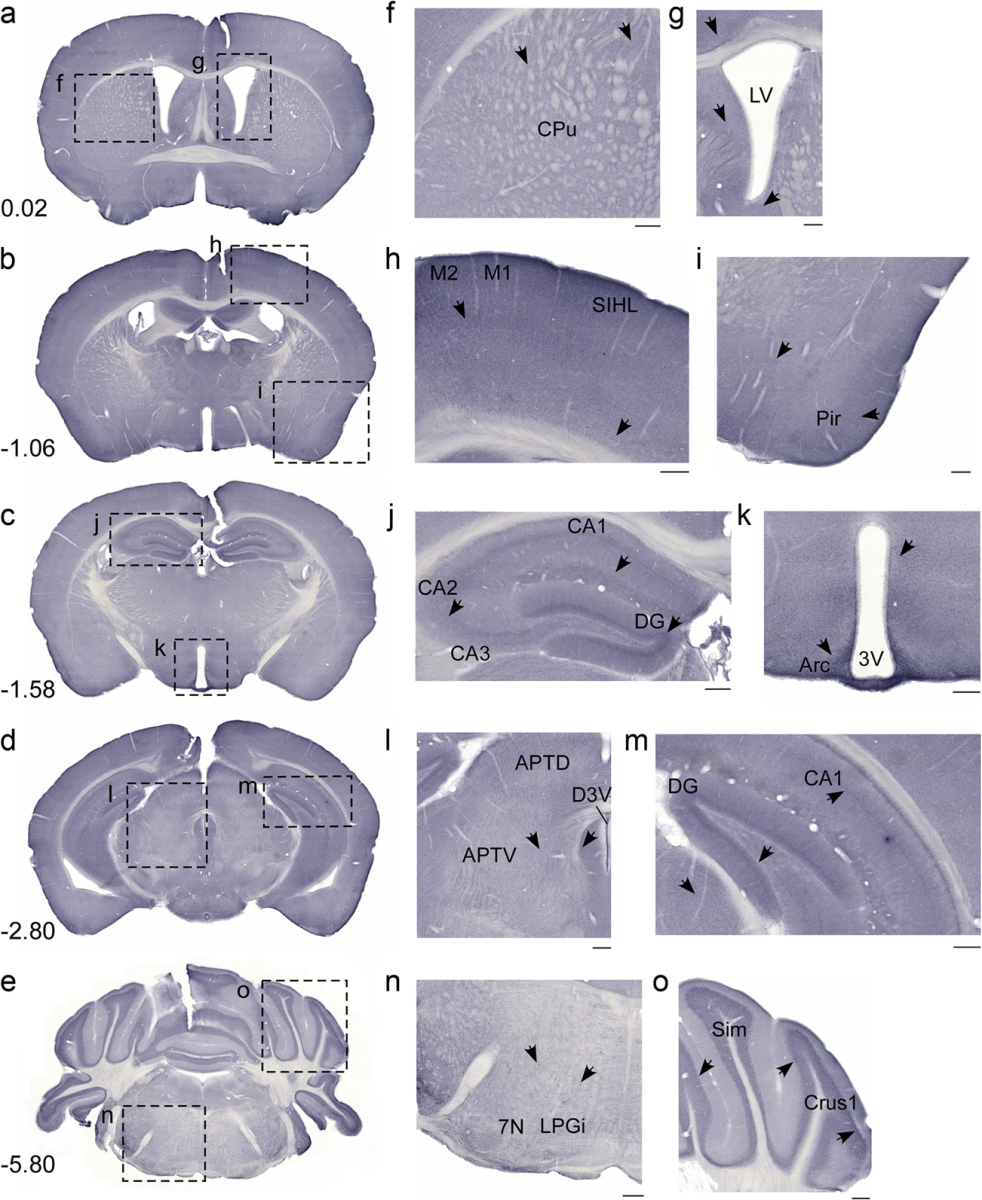
Bright field MFSD3 protein screening in coronal mouse brain sections. MFSD3 staining on free-floating sections displayed a homogenous staining pattern. Here are overview pictures (**a**–**e**) and enlarged images with *scale bars* of 200 μm (**f**–**o**) shown. (**a**) Bregma 0.02 mm, (**b**) Bregma −1.06 mm, (**c**) Bregma −1.58 mm, (**d**) Bregma −2.80 mm and (**e**) Bregma −5.80 mm display the overall staining, including dashed lines that indicates the magnifications. Staining was located to at the (**f**) caudate putamen and (**g**) around the lateral ventricle; (**h**) motor and primary somatosensory areas and (**i**) piriform cortex; (**j**) the hippocampus, fields CA1, CA2 and CA3 and dentate gyrus, and (**k**) arcuate hypothalamic nuclei around the third ventricle. Staining was also found around the dorsal third ventricle, in the area anterior pretectal nucleus area (**l**). Staining was also seen in midbrain areas and (**m**) the dentate gyrus. Finally, cells were seen in the brainstem, e.g. in the facial nucleus (**n**), and in the cerebellum (**o**), e.g. Crus 1 area

Non-fluorescent immunohistochemistry on 70-μm free-floating coronal mouse brain sections was performed using the anti-MFSD1 (Fig. 5, overview pictures (a–e) and close-up pictures (f–o)) and the anti-MFSD3 antibodies (Fig. 6, overview pictures (a–e) and close-up pictures (f–o)). MFSD1 staining was seen in cortex, striatum hippocampus, hypothalamus, thalamus, brainstem and cerebellum. Based on morphology, MFSD1 labelled various parts of the cell body and different cell types. As seen in the magnified images, the MFSD1 antibody stained cerebral cortex areas such as piriform cortex (Fig. 5f) and primary somatosensory barrel (Fig. 5h). Staining was also seen in striatum (Fig. 5g), thalamic nuclei (Fig. 5i) and the arcuate hypothalamic nucleus (Fig. 5j). In addition to the soma staining, MFSD1 labelled projections seen in the hippocampus (Fig. 5k, m) and cells in midbrain areas like the red nucleus (Fig. 5l), together with cells in brainstem areas such as the facial nucleus (Fig. 5n). Also, the Purkinje cell layer in cerebellum was stained by the MFSD1 antibody (Fig. 5o).

Immunostaining with the MFSD3 antibody was more homogenous and abundant than MFSD1 staining. MFSD3 staining was located in, e.g. cortex, striatum, hippocampus, hypothalamus, thalamus, cerebellum (Fig. 6a–e); specifically found in striatum, caudate putamen (Fig. 6f) and in the area around the lateral ventricles (Fig. 6g). There was also homogenous staining throughout cerebral cortex (Fig. 6h) and piriform cortex (Fig. 6i). In addition, staining was located in all hippocampus fields, together with dentate gyrus (Fig. 6j, m). Hypothalamic staining was detected in the areas around the third ventricle, with high expression in arcuate hypothalamic nucleus (Fig. 6k), as well as in midbrain areas lateral of the midline (Fig. 6l) and in the brainstem (Fig. 6n). Finally, cerebellar staining was detected in for example the Crus 1 area (Fig. 6o).

### Fluorescent Co-staining Reveals Specific Subcellular Neuronal Staining

**Fig. 7:**
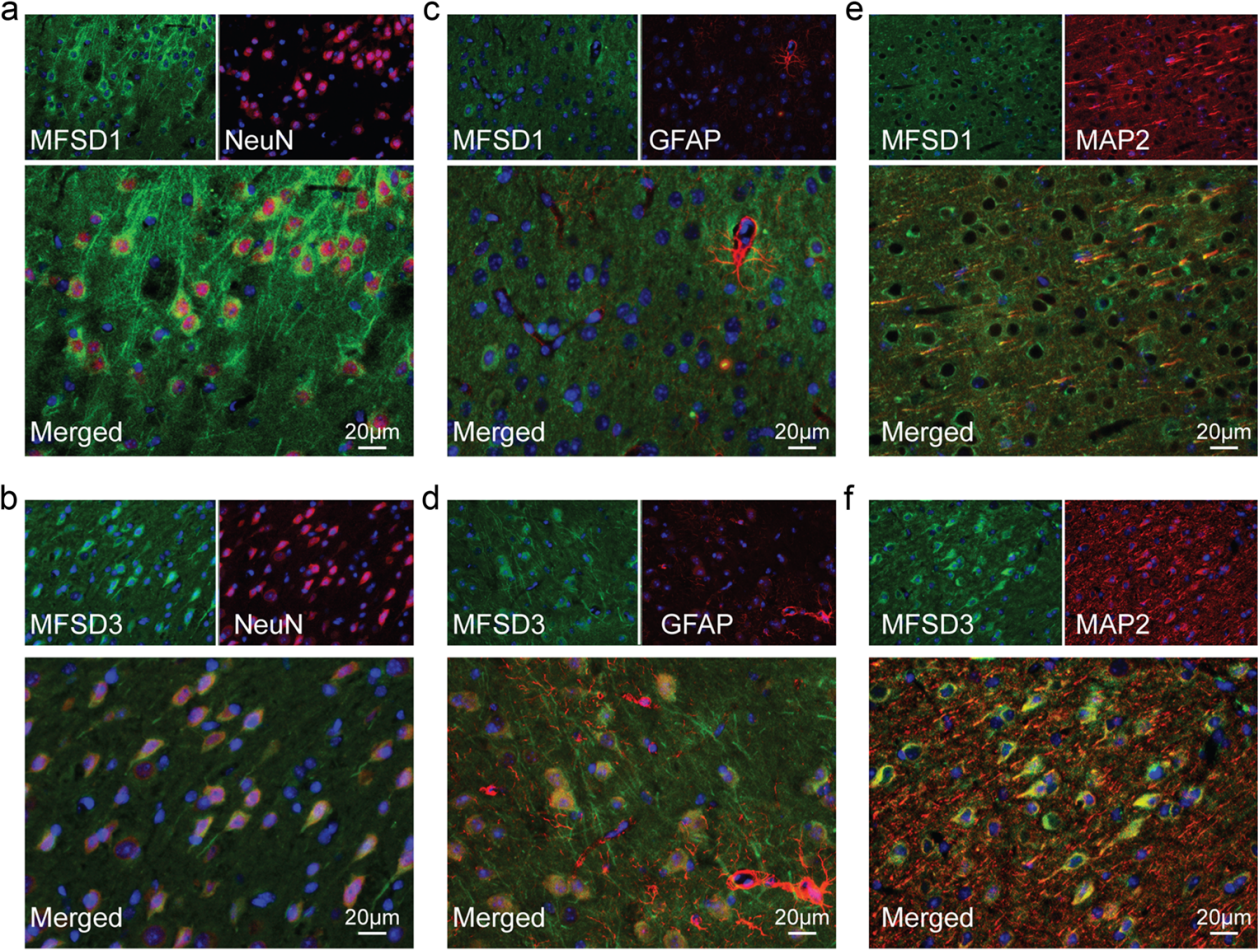
MFSD1 and MFSD3 are expressed specifically in neurons. To determine cell type expression, fluorescent immunostaining on coronal mouse brain sections, with nuclear DAPI staining were run. This revealed co-staining between MFSD1 (**a**) and MFSD3 (**b**), with the neuronal marker NeuN as seen in the merged images. No overlap could be seen with either the astrocytic marker GFAP (**c**, **d**) or the dendritic marker MAP2 (**e**, **f**). Micrographs were acquired using a fluorescence Olympus microscope BX53 with an Olympus DP73 camera

**Fig. 8:**
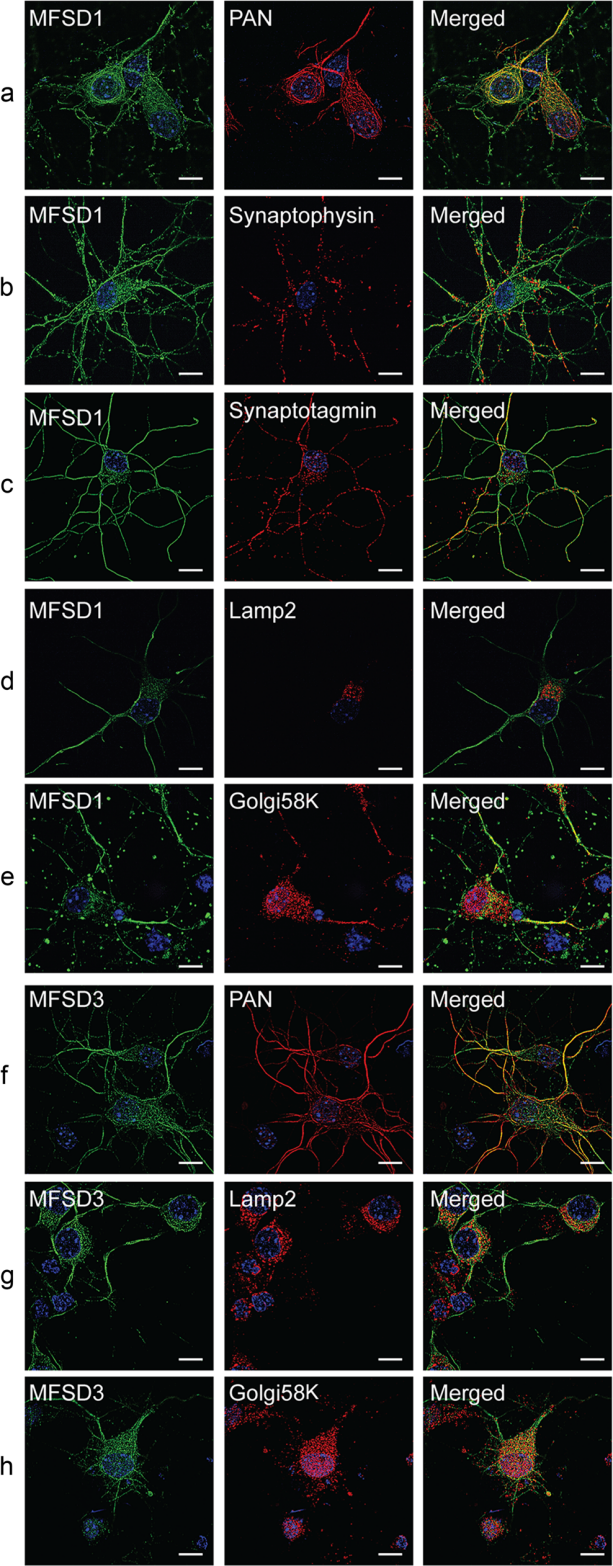
Subcellular localization of MFSD1 and MFSD3. The subcellular distribution of MFSD1 and MFSD3 was studied using fluorescent immunostaining in mouse embryonic primary cortex cells. MFSD1 co-localized well with PAN (**a**) and partial overlap with synaptophysin (**b**) and synaptotagmin (**c**) was detected. No co-staining was seen with Lamp2 (**d**) or Golgi58K (**e**). MFSD3 co-localized with PAN (**f**), but not with Lamp2 (**g**) or Golgi58K (**h**). Micrographs were acquired using an LSM710 super resolution microscope. *Scale bars* represent 10 μm

To study in what cell types MFSD1 and MFSD3 were expressed, they were co-labelled with cellular markers on mouse brain sections. Staining revealed overlap with the neuronal marker NeuN (Fig. 7a, b), but not with the astrocytic marker GFAP (Fig. 7c, d) or the dendritic marker MAP2 (Fig. 7e, f). The subcellular location of the MFSD1 and MFSD3 was studied in embryonic primary cortex cells using immunocytochemistry. The anti-MFSD1 antibody co-stained with PAN (Fig. 8a) in a subset of neurons. Partial overlap with the vesicular marker synaptophysin (Fig. 8b) and the synaptic vesicle marker synaptotagmin (Fig. 8c) were observed, suggesting co-expression in synapses. No overlap was detected with the lysosome marker Lamp2 (Fig. 8d) or the Golgi marker Golgi58K (Fig. 8e). For MFSD3, co-staining was detected with PAN (Fig. 8f), but not with the Lamp2 (Fig. 8g) or the Golgi58K marker (Fig. 8h).

### Gene Expression Following Amino Acid Deprivation in Mouse Embryonic Cortex Cells

**Fig. 9:**
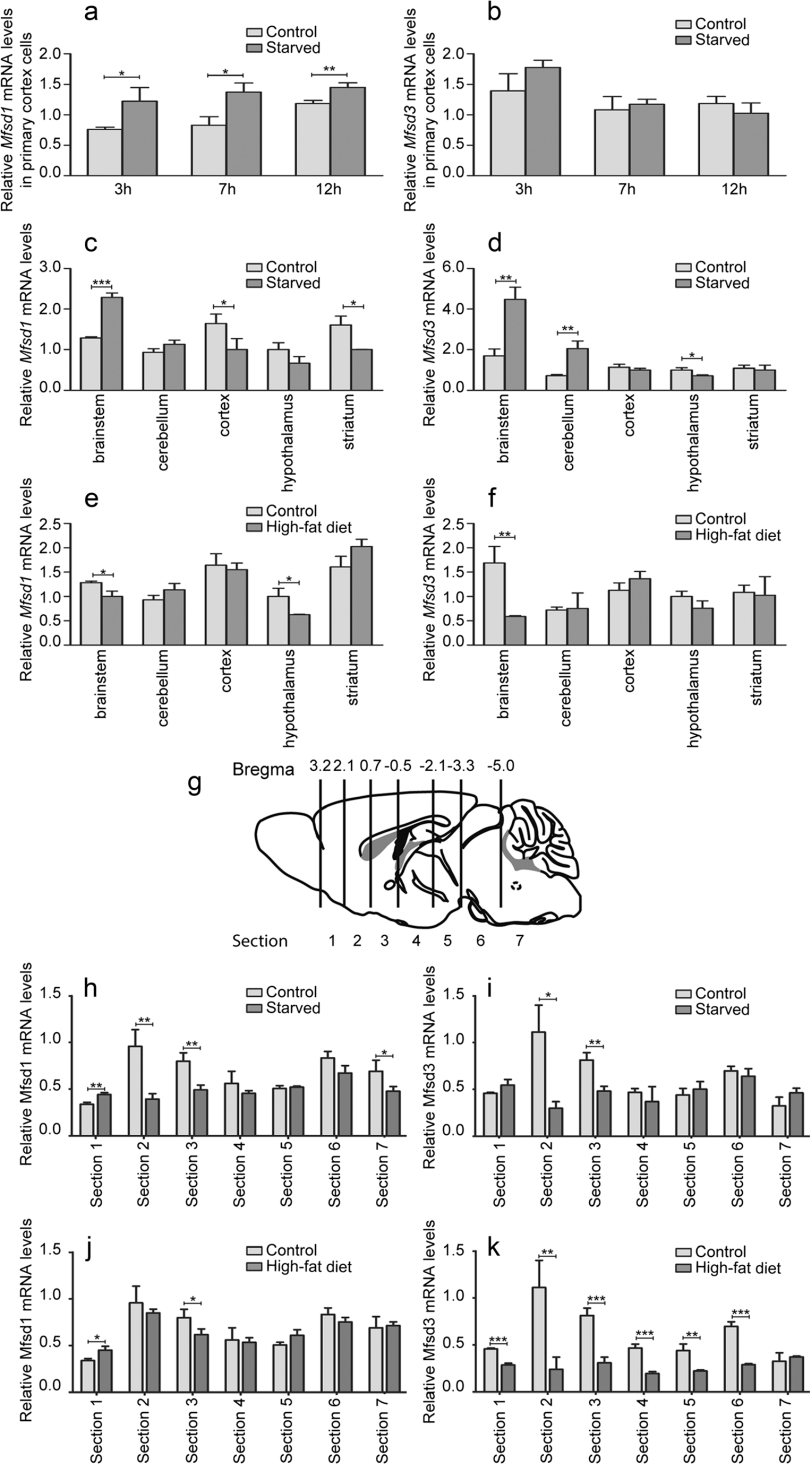
mRNA expression changes after altered energy intake in primary cells and the mouse brain. Primary mouse embryonic cortex cells were deprived of nine amino acids for 3, 7, or 12 h while the control cells had access to these amino acids. The gene expression for *Mfsd1* (**a**) and *Mfsd3* (**b**) was measured. The graphs display the relative mRNA expressions (±SD), (*p ≤ 0.05, **p ≤ 0.01 and ***p ≤ 0.001). Mice were assigned tree different food paradigms before euthanasia; (1) normal chow (control), (2) 24-h starvation and (3) high-fat diet (HFD). The gene expression was measured for *Mfsd1* and *Mfsd3* in brainstem, cerebellum, cortex, hypothalamus and striatum after starvation (**c**, **d**) and HFD (**e**, **f**) where *n* = 4 per group. Brains from additional mice (controls, *n* = 6, starved *n* = 4 and HFD *n* = 6) were excised into seven sections (**g**), image adjusted from (Perland et al. 2016). Expression levels after starvation are depicted **h** for *Mfsd1* and **i** for *Mfsd3* and after HFD for *Mfsd1* (**j**) and *Mfsd3* (**k**). *Gapdh*, *H3a* and *Actb* were used as reference genes. *p ≤ 0.0493, **p ≤ 0.00998 and ***p ≤ 0.001 after Bonferroni correction

Mature mouse embryonic cortex cells were cultured in limited or complete amino acid medium for 3, 7, or 12 h, followed by gene expression measurements for *Mfsd1* and *Mfsd3*. Following amino acid limitation, *Mfsd1* was upregulated at 3 h (*p* = 0.0249), 7 h (*p* = 0.0103) and 12 h (*p* = 0.0077) of treatment compared with amino acid-treated control cells (Fig. 9a) while *Mfsd3* was not altered at any time (3 h (*p* = 0.0950), 7 h (*p* = 0.5418) and 12 h (*p* = 0.2499)) (Fig. 9b).

### Gene Expression after Changed Energy Homeostasis

As altered expressions were detected after amino acid starvation in cortex cells, we found it interesting to study it in vivo as well. The gene expressions were measured in cortex and other brain areas, from mice following starvation and HFD. We included brainstem, cerebellum, cortex, hypothalamus and striatum in the analyses. After 24-h starvation, *Mfsd1* was upregulated in brainstem (*p* = 0.0001) while downregulated in cortex (*p* = 0.0358) and striatum (*p* = 0.0333) (Fig. 9c) compared with controls. *Mfsd3* was upregulated in brainstem (*p* = 0.0064) and cerebellum (*p* = 0.0037) and downregulated in hypothalamus (*p* = 0.00145) (Fig. 9d). Following HFD, *Mfsd1* was downregulated in brainstem (*p* = 0.0124) and hypothalamus (*p* = 0.0181) (Fig. 9e) and *Mfsd3* was downregulated in brainstem (*p* = 0.0049) (Fig. 9f).

As interesting effects were measured in the brain areas, new mice were exposed to starvation and HFD. These brains were sectioned according to the schematic brain (Fig. 9g), adapted from (Perland et al. 2016). Both genes responded in similar fashion to starvation with downregulation in sections 2 (*Mfsd1 p* = 0.0067; *Mfsd3 p* = 0.0207) and 3 (*Mfsd1 p* = 0.0067; *Mfsd3 p* = 0.0037) (Fig. 9h, i). In addition, *Mfsd1* was upregulated in section 1 (*p* = 0.0035) and downregulated in section 7 (*p* = 0.047) (Fig. 9h) after starvation. In the obese mice, the mRNA levels of *Mfsd1* were upregulated in section 1 (*p* = 0.0127) compared with the controls and downregulated in section 3 (*p* = 0.0442) (Fig. 9j), whereas the expression of *Mfsd3* was reduced in all but section 7; sections 1 (*p* = 0.0002), 2 (*p* = 0.009), 3 (*p* = 0.0009), 4 (*p* = 0.0005), 5 (*p* = 0.006) and section 6 (*p* = 0.0002) (Fig. 9k).

## Discussion

In this work, we identified and characterized two novel proteins, MFSD1 and MFSD3, and presented their histological expression pattern in the mouse brain, providing a solid ground for further work to be based on. The fact that they are named according to the MFSD nomenclature suggests that they are atypical SLCs (Fredriksson et al. 2008) and hence possibly transporters. Both proteins also belong to the major facilitator superfamily (MFS), which is the largest group of facilitators and secondary active transporters (Law et al. 2008; Pao et al. 1998; Reddy et al. 2012; Saier et al. 1999) in several phylum (Pao et al. 1998). As MFSD1 and MFSD3 contain MFS motifs, we performed a phylogenetic analysis showing their similarity to SLCs of MFS type and other MFSD proteins, strengthening the hypothesis that they are indeed transporters. The HUGO Gene Nomenclature Committee (HGNC) classifies SLCs into families based on function, homology or phenotype (Gray et al. 2016), where family members share 20 % sequence identity with other members (Hediger et al. 2004). This means that neither MFSD1 nor MFSD3 can be classified into their phylogenetically closest SLC family, as investigated here. However, SLC29A4 clustered phylogenetically more closely to MFSD3 than to its own family, SLC29 (Fig. 1a), while it shared highest sequence identity with SLC29 members. This means that MFSD1 and MFSD3 could still share more than 20 % sequence identity to other SLC members and hence belong to that SLC family.

Based on the secondary and tertiary structures, we predicted MFSD1 and MFSD3 to have 12 TMS each, which is common for MFS proteins (Law et al. 2008), as they all derived from the same evolutionary origin (Reddy et al. 2012). An interesting note is that SLC29 members, the closest to MFSD1, are predicted to have 11 TMS regions (Baldwin et al. 2004; Valdes et al. 2014; Young et al. 2008), even though they are SLCs of MFS type, which could explain why the sequence identities to *Mfsd1* were relatively low. As phylogenetically related SLCs generally share similar substrates (Schlessinger et al. 2010), it is plausible that MFSD1 and MFSD3 transport organic substrates since they clustered with SLCs having organic substrates. MFSD3 is a predicted Acetyl-CoA transporter according to Kyoto Encyclopaedia of Genes and Genomes (Kanehisa et al. 2016), and we showed that it shares evolutionary origin and relatively high sequence identity with SLC33A1, a known Acetyl-CoA transporter (Hirabayashi et al. 2013), enhancing the hypothesis of organic substrate profiles. However, preferable substrates for MFSD1 and MFSD3 still need to be elucidated.

The homology models of MFSD1 and MFSD3 enforce the possibility that they are permeases. Both the C- and N-terminals are predicted to be located in the cytosolic side of the membrane, which is in accordance with previous reports regarding MFS proteins (Yan 2013). Also, when looking at the 3D structures, both seem to fold into a cylinder (seen in Fig. 3b, d), through which a potential substrate could be shuffled. However, based on global model quality estimation (GMQE), the MFSD1 and MFSD3 models both scored around 0.5 on the 0–1 scale, where higher values indicates a more probable model. Therefore, it is important to remember that these are predications and the actual structures still need to be determined.

With fluorescent double-staining, we demonstrated that both proteins were specifically expressed in neurons within the mouse brain. When looking at the MFSD1 staining, neuronal projections were labelled; presumably, they were axonal projections as no co-staining was detected with the dendritic microtubule marker MAP2. In the primary cells, MFSD1 overlapped with the neuronal marker PAN in a subset of neurons. There was also partial overlap with the vesicular markers synaptophysin and synaptotagmin, which is possible if MFSD1 is located in the plasma membrane. If the epitopes for the antibodies are in close proximity to each other, it is likely to see colour overlay. However, as there was no complete overlap, we found it most likely that MFSD1 was present along the plasma membrane and not constricted to the active zones. Interestingly, no overlap with the lysosomal marker Lamp2 was detected, as previously suggested in stained HeLa cells (Palmieri et al. 2011). Some overlap signal could be seen between the MFSD1 and Lamp2 markers before performing the super resolution processing. However, when looking through the images in the Z-stack, the colours were located in different focus points in the cells. No colour overlay was detected in the final image because MFSD1 expression was probably in the plasma membrane while Lamp2 was intracellular.

MFSD3 had a broader staining pattern than MFSD1 and was located in the membrane in most neurons. Note that anti-MFSD3 also bound epitopes having similar intracellular morphology as the Golgi marker; however no significant overlay between the markers was detected looking through the Z-stacks. As proteins are being processed for further transport in Golgi, it is possible to get staining there, even though the Golgi is not the final location of the protein. Therefore, it is most likely that MFSD3 is functionally active in the plasma membrane and not in any intracellular organelles.

It has previously been reported that several genes encoding characterized amino acid transporters from the SLC superfamily, are upregulated following amino acid deprivation in cells, e.g. *Slc38a2* (Palii et al. 2006), *Slc7a1* (Fernandez et al. 2003), *Slc7a5* (Padbury et al. 2004), *Slc7a11* (Sato et al. 2004), *Slc1a4* and *Slc3a2* (Lee et al. 2008). However, not all amino acid transporters are regulated in response to amino acid starvation and the increased expression levels detected for *Mfsd1* do not necessarily mean that it is an amino acid transporter. MFSD1 is a predicted sugar transporter (Kanehisa et al. 2016), and both glutamine (Nurjhan et al. 1995) and alanine (Felig et al. 1970) can be constituents in glucose synthesis. Hence, also transporters with substrates that are synthesized from amino acids can be transcriptionally upregulated. However, even though MFSD3 is a predicted acetyl-CoA transporter (Kanehisa et al. 2016) and acetyl-CoA has amino acids as precursors (Arrieta-Cruz and Gutierrez-Juarez 2016), it was un-altered by the partial amino acid deprivation.

Half of all *Slc* genes are present in areas involved in energy production and food consumption (Dahlin et al. 2009), as were the case for MFSD1 and MFSD3. Looking at in vivo effects in brain areas involved in metabolism, following changed nutritional status, it indicated that expression of both genes was affected. *Mfsd1* was downregulated specifically in cortex and striatum after starvation, which are both well-known areas involved in food and reward pathways (Farr et al. 2016). *Mfsd1* reduction was also seen in three of the brain sections; section 2 that included cortex, section 3, with e.g. the striatum and section 7 with cerebellum and brainstem. Interestingly, after starvation, *Mfsd1* and *Mfsd3* were specifically upregulated in brainstem, that gate transmitting signals from peripheral circulating hormones to the remaining brain (Mikulaskova et al. 2016). Hence, the downregulation of *Mfsd1* in section 7 could be due to effects in cerebellum, possibly the cerebellar-hypothalamic circuits which convey signals from both hormones and gastric vagal afferents to hypothalamic neurons (Zhu and Wang 2008). Both genes responded similarly to food deprivation, but, after high-fat consumption, *Mfsd1* was stable, while *Mfsd3* was reduced extensively. Even if the two transporters at first seem similar, with common ancestral back ground, and expression in similar brain areas, they probably have different roles in vivo.

For the in vivo study, we included specific brain areas and larger brain sections. Since we know that *Mfsd1* and *Mfsd3* were specifically expressed in a subpopulation of cells, it is possible that specific changes are diluted in the larger sample. Analyses of brain sections are a good way to include whole circuits as neurons can project beyond specific areas or nuclei. Therefore, we found it interesting to study changes in mRNA expression in both specific brain areas and larger brain sections, to get the best representation of actual functions.

We used the C57BL/6J mouse strain for the in vivo energy homeostasis study since the mice responds in similar way as humans regarding the metabolic syndrome (Collins et al. 2004). Therefore, there is the possibility that the measurable effects after both starvation and HFD are due to secondary effects such as stress and inflammation. However, it seems that SLCs involved in the immune response are more frequently induced during inflammation (Cellier 2013; Sasawatari et al. 2011), whereas both *Mfsd1* and *Mfsd3* were reduced. Hence, we found it most likely that the effects are primarily due to food rather than inflammation or stress.

In conclusion, we have studied MFSD1 and MFSD3, two novel atypical SLCs that phylogenetically clustered with SLCs of MFS type. We displayed their abundant mRNA expression and their location to the plasma membrane in neurons. Finally, we demonstrated that *Mfsd1* and *Mfsd3* mRNA levels were affected by altered nutrition intake.
